# Association between 25-Hydroxyvitamin D Status and Components of Body Composition and Glucose Metabolism in Older Men and Women

**DOI:** 10.3390/nu10121826

**Published:** 2018-11-25

**Authors:** Svea-Vivica Mathieu, Karina Fischer, Bess Dawson-Hughes, Gregor Freystaetter, Felix Beuschlein, Simeon Schietzel, Andreas Egli, Heike A. Bischoff-Ferrari

**Affiliations:** 1 Department of Geriatrics and Aging Research, University Hospital Zurich, 8091 Zurich, Switzerland; svea-vivica.mathieu@uzh.ch (S.-V.M.); karina.fischer@uzh.ch (K.F.); gregor.freystaetter@usz.ch (G.F.); simeon.schietzel@usz.ch (S.S.); andreas.egli@usz.ch (A.E.); 2 Centre on Aging and Mobility, University Hospital Zurich, 8091 Zurich, Switzerland; 3 Jean Mayer USDA Human Nutrition Research Center on Aging, Tufts University, Boston, MA 02153, USA; bess.dawson-hughes@tufts.edu; 4 Department of Endocrinology, Diabetes, and Clinical Nutrition, University Hospital Zurich, 8091 Zurich, Switzerland; felix.beuschlein@usz.ch; 5 University Clinic for Acute Geriatric Care, City Hospital Waid, 8037 Zurich, Switzerland

**Keywords:** ageing, elderly, body composition, diabetes, insulin resistance, metabolic syndrome, vitamin D

## Abstract

Obesity and sarcopenia are major causes of morbidity and mortality among seniors. Vitamin D deficiency is very common especially among seniors and has been associated with both muscle health and obesity. This study investigated if 25-hydroxyvitamin D (25(OH)D) status is associated with body composition and insulin resistance using baseline data of a completed RCT among relatively healthy community-dwelling seniors (271 seniors age 60+ years undergoing elective surgery for unilateral total knee replacement due to osteoarthritis). Cross-sectional analysis compared appendicular lean mass index (ALMI: lean mass kg/height m^2^) and fat mass index (FMI: fat mass kg/height m^2^) assessed by DXA and insulin resistance between quartiles of serum 25(OH)D concentration using multivariable linear regression adjusted for age, sex, smoking status, physical activity, and body mass index (BMI). Participants in the lowest serum 25(OH)D quartile (4.7–17.5 ng/mL) had a higher fat mass (9.3 kg/m^2^) compared with participants in the third (8.40 kg/m^2^; Q3 = 26.1–34.8 ng/mL) and highest (8.37 kg/m^2^; Q4 = 34.9–62.5 ng/mL) quartile (*p*_overall_ = 0.03). Higher serum 25(OH)D quartile status was associated with higher insulin sensitivity (*p*_overall_ = 0.03) and better beta cell function (*p* = 0.004). Prevalence of insulin resistance tended to be higher in the second compared with the highest serum 25(OH)D quartile (14.6% vs. 4.8%, *p* = 0.06). Our findings suggest that lower serum 25(OH)D status may be associated with greater fat mass and impaired glucose metabolism, independent of BMI and other risk factors for diabetes.

## 1. Introduction

Age-related loss of strength and muscle mass coupled with higher body fat mass contribute to mobility disability and frailty at an older age [1]. In its extremes, loss of muscle mass and relative gain in fat mass has been conceptualized as sarcopenic obesity [2], meaning the concomitant presence of sarcopenia and obesity coupled with insulin resistance [3]. The prevention of both conditions, namely shifting the population towards a healthier body composition, may have enormous public health benefits by decreasing mobility disability and frailty [4], and many contributing co-morbid conditions [5].

Supplementation of vitamin D may be a simple, safe, and cost-effective strategy to support a healthier body composition at an older age. Vitamin D deficiency is very common among seniors [6] and has been linked to both low muscle mass [7] and high fat mass [8,9] as well as metabolic disturbances such as insulin resistance [10].

Mechanistically, the vitamin D receptor (VDR) is expressed in muscle tissue [11,12,13], and its activation results in de novo muscle protein synthesis, as confirmed in a randomized placebo-controlled clinical trial among postmenopausal women [14]. The VDR has also been found in fat tissue [15], and in-vitro experiments have suggested a vitamin D-induced increase of intracellular calcium in adipocytes and thereby a decrease in lipogenesis and increase in lipolysis [16].

Also, at a clinical level, vitamin D has been linked to better muscle health and glucose metabolism [17,18]. Vitamin D deficiency has been associated with muscle mass loss [19,20], proximal muscle weakness [21], and impaired lower extremity function [22]. Further, vitamin D deficiency has been linked to higher insulin resistance [10], lower insulin sensitivity, earlier feelings of hunger, and higher food intake [23].

Despite these earlier findings, to our knowledge, data on the study of muscle mass, fat mass, and insulin resistance in relation to 25-hydroxyvitamin D (25(OH)D) status in one population of seniors is missing. Therefore, the aim of the present study was to investigate the association between serum 25(OH)D concentration and fat mass, muscle mass, as well as insulin resistance in relatively healthy community-dwelling senior men and women undergoing elective surgery for severe unilateral knee osteoarthritis.

## 2. Materials and Methods

### 2.1. Study Design and Population

The present study is a secondary, cross-sectional observational analysis using baseline data from the Zurich Multiple Endpoint Vitamin D Trial in Knee Osteoarthritis Patients (NCT00599807; clinicaltrials.gov), a 2-year double-blind randomized controlled trial that tested two different vitamin D supplementation regimens (2000 vs. 800 IU/day cholecalciferol) in senior knee osteoarthritis patients. In brief, 273 patients age 60 years and older were enrolled 6–10 weeks after undergoing unilateral total knee replacement due to severe knee osteoarthritis. Baseline measurements took place 6–10 weeks after surgery at the Centre on Aging and Mobility at the University of Zurich, Switzerland, from October 2007 to February 2013. Most important exclusion criteria for the present analysis included history of chronic corticosteroid use, history of malabsorption disorder, current cancer, and inability to walk at least 3 m with or without a walking aid. The protocol of the clinical trial was approved by the Cantonal Ethical Commission of Zurich, Switzerland (Protocol identifier STZ 20/07). The study was in accordance with the principles as outlined in the Declaration of Helsinki, and all participants gave written informed consent. For the present study, two participants were excluded due to missing information on serum 25(OH)D concentration, resulting in an analytical sample size of 271 participants.

### 2.2. Assessment of Serum 25(OH)D Concentration

Fasting blood samples were taken in the morning when the participants arrived at the study center. Serum 25(OH)D concentration was measured using liquid chromatography-tandem mass spectrometry-based multiple reaction monitoring (LC-MS/MS MRM, coefficient of variation of ±15%) [24,25] performed by DSM Nutritional Products Ltd., R & D Analytics, Quantitative Bioanalytics (Basel, Switzerland) and included in the NIST/NIH Vitamin D Metabolites Quality Assurance Program [26]. Specifically, separation and quantification were performed on an Agilent 1290 ultra-high-performance liquid chromatography (Agilent Technologies, Santa Clara, CA, USA) coupled with an API 4000 mass spectrometer (AB Sciex, Framingham, MA, USA) using multiple reaction monitoring transitions. Serum 25(OH)D concentrations are stated in ng/mL with 1 ng/mL being equivalent to 0.4 nmol/L. According to local specifications, serum 25(OH)D concentrations <20 ng/mL were considered as deficiency and <10 ng/mL as severe deficiency.

### 2.3. Assessment of Participant Characteristics and Covariates

Body weight (BW, kg) and height (cm), as well as age, sex, and smoking status (non, past, and current smokers), were assessed by questionnaire. We calculated body mass index (BMI) by dividing body weight by height squared (kg/m^2^). Physical activity was assessed objectively using an ankle-worn ambulatory activity monitor (StepWatch™ Step Activity Monitor, Cyma, Seattle, WA, USA), which records the number of steps taken every minute. The StepWatch™ monitor has been validated for the use in older adults [27] and has been used to monitor physical activity in several patient groups including patients with knee osteoarthritis [28]. Participants were asked to wear the monitor during wakeful hours for seven consecutive days. We considered a measurement as valid if at least three days with ≥10 h of recording were available, omitting blocks of >180 min of consecutive zeros, which was interpreted as device not worn. Minutes spent in moderate-to-vigorous physical activities (MVPA) were defined as the average minutes per day with ≥30 steps/min according to the manufacturer’s software manual (StepWatch™ 3.1 Software Manual).

### 2.4. Assessment of Measures of Insulin Resistance

Fasting plasma blood concentration of glucose was measured in fluoride plasma by enzymatic reference method with hexokinase using the MODULAR P system analyzer (coefficient of variation of 1.7% at 5.4 mmol/L and 1.2 at 12.6 mmol/L) from Roche/Hitachi (Roche Diagnostics AG, Rotkreuz, Switzerland). Fasting serum blood concentration of insulin was measured in serum by chemiluminescence enzyme immunoassay using Immulite 2500 (CV of 5.7% at 88.8 pmol/L and 3.2% at 914.9 pmol/L) from Diagnostic Products Corporation (Siemens Diagnostic AG, Zurich, Switzerland). All these blood analyses were performed at the Institute of Clinical Chemistry, University Hospital Zurich, Switzerland. We used the updated homeostasis model assessment (HOMA2) with a threshold of ≥1.8 for insulin resistance [29] to estimate parameters of glucose metabolism: insulin resistance as both a continuous and a binary variable (HOMA2-IR, index value, the lower the better), beta cell function (HOMA2%B, percentage, the lower the better), and insulin sensitivity (HOMA2%S, percentage, the higher the better).

### 2.5. Assessment of Body Composition

Total and appendicular lean mass as well as total fat mass were measured by dual-energy X-ray absorptiometry (DXA) with a coefficient of variation of 0.78% for fat mass and 0.52% for lean mass (Hologic QDR 4500A fan-beam densitometer; Hologic, Inc., Bedford, MA, USA; Hologic Discovery version 12.4 software). From these measurements, lean mass index (LMI), appendicular lean mass index (ALMI), and fat mass index (FMI) were calculated by the following equations:

ALMI = appendicular lean mass [kg]/ height [m]^2^ (1)

FMI = fat mass [kg]/ height [m]^2^ (2)

LMI = lean mass [kg]/ height [m]^2^ (3)

### 2.6. Statistics

Statistical analysis was performed using SAS 9.4 (SAS Institute, Inc., Cary, NC, USA). Distributions of continuous variables were examined for normality. To analyze differences between men and women, we used Student’s *t* test for continuous variables and a χ^2^ test for categorical variables.

Multivariable-adjusted analysis of covariance (ANCOVA) models were used to compare least-square mean values (LSM) of the outcome variables ALMI, FMI, fasting blood glucose, fasting blood insulin, HOMA2-IR, HOMA2%B, and HOMA2%S between quartiles of serum 25(OH)D concentration in the total population or stratified by sex. All models were adjusted for BMI, age, sex, smoking status, and physical activity; except for the model of FMI (which was adjusted for age, sex, smoking status, LMI, and physical activity) and ALMI (which was adjusted for age, sex, smoking status, FMI, and physical activity). Linear regression analysis was performed to test for a linear trend across the serum 25(OH)D quartiles by using the median values of each quartile as a continuous variable. One participant with an outlying ALMI value (>2 standard deviations above the mean ALMI) was excluded from all analyses on the ALMI.

Generalized linear models (using the logit link function) unadjusted and adjusted for age, sex, physical activity, smoking status, and BMI were used to analyze differences in the prevalence of insulin resistance (HOMA2 IR ≥1.8) and diabetes (fasting blood glucose >7 mmol/L) between serum 25(OH)D quartiles.

Notably, as all endpoints showed the same pattern for men and women, all results are presented for the total population adjusted inter alia for sex.

Statistical significance was set at *p* < 0.05 with reported *p* values being two-sided.

## 3. Results

### 3.1. Participants’ Characteristics

Participants’ characteristics (53.5% women; mean age 70.3 ± 6.4 years) are presented by sex (Table 1). Mean serum 25(OH)D concentration of the total population was 27.3 ± 12.4 ng/mL with 85 (31.4%) participants being vitamin D deficient (<20 ng/mL). Mean serum 25(OH)D levels did not differ significantly between men and women. Quartiles of serum 25(OH)D concentration (ng/mL) among 271 participants were as follows: 4.7–17.5 (quartile 1), 17.6–26.0 (quartile 2), 26.1–34.8 (quartile 3), 34.9–62.5 (quartile 4). Men were taller, heavier, more likely to be overweight, and more physically active (45.1 ± 22.8 vs. 38.0 ± 21.2 min MVPA/day; *p* = 0.009) than women and were also more frequently current or past smokers than women. Women had higher fat mass (37.7% vs. 26.7%; *p* < 0.0001), less total lean mass (60.0% vs. 70.0%; *p* < 0.0001), and less appendicular muscle mass (25.3% vs. 31.0%; *p* < 0.0001) than men. Moreover, fasting blood glucose (5.3 vs. 5.9 mmol/L; *p* < 0.0001) and insulin (7.0 vs. 9.0 mU/L; *p* = 0.03) concentration as well as insulin resistance (0.9 vs. 1.2 HOMA2 IR; *p* = 0.005) and prevalence of diabetes (0.0% vs. 8.0%; *p* = 0.0004) were significantly lower, whereas insulin sensitivity (195.7 vs. 143.3 HOMA2%S; *p* = 0.0005) was significantly higher among women than men.

### 3.2. Association between Quartiles of Serum 25(OH)D and Fat Mass (FMI)

**Figure 1:**
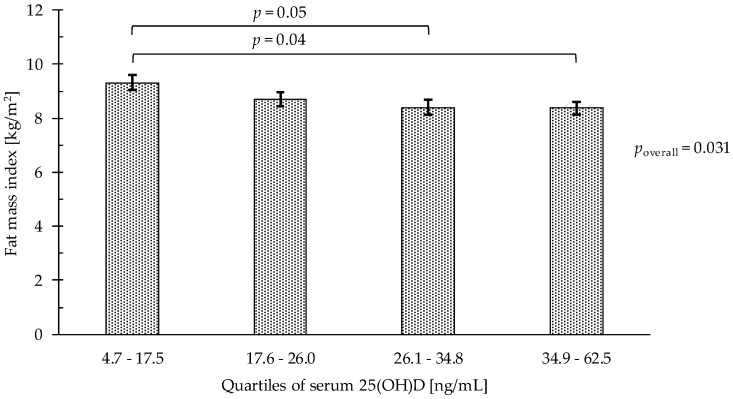
Fat mass index by serum 25(OH)D quartiles (*n* = 271), 1 ng/mL 25-hydroxyvitamin D being equivalent to 0.4 nmol/L. Bars represent least-square means (with whisker for standard error) from multivariable linear regression models. Models were adjusted for age, sex, MVPA, smoking status (non, past, and current smoker) and LMI. 25(OH)D, 25-hydroxyvitamin D; LMI, lean mass index; MVPA, moderate to vigorous physical activity (min/day).

For fat mass (Table 2, Figure 1), adjusting for age, sex, smoking status, LMI, and physical activity, the FMI of participants in the lowest serum 25(OH)D quartile was higher compared with participants in the third (9.3 kg/m^2^ vs. 8.4 kg/m^2^; *p* = 0.049) and highest (9.3 kg/m^2^ vs. 8.4 kg/m^2^; *p* = 0.04) quartile.

### 3.3. Association between Quartiles of Serum 25(OH)D and Muscle Mass (ALMI)

For muscle mass (Table 2), adjusting for age, sex, smoking status, FMI, and physical activity, the ALMI was not associated with quartiles of serum 25(OH)D.

### 3.4. Association between Quartiles of Serum 25(OH)D and Glucose Metabolism

For fasting insulin concentration (Table 3), adjusting for age, sex, smoking status, BMI, and physical activity, insulin concentration did not differ significantly between quartiles of serum 25(OH)D.

**Figure 2:**
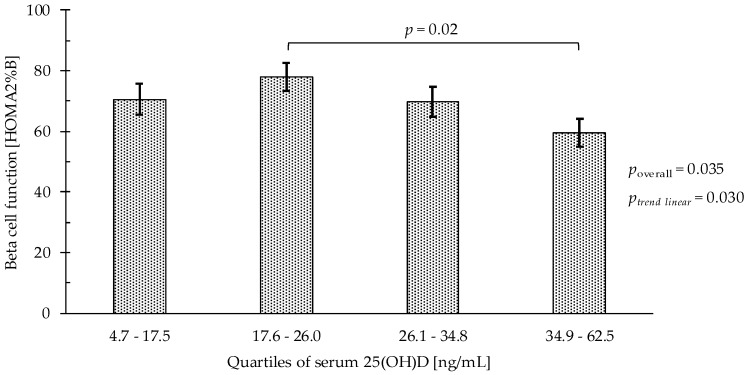
Beta cell function (HOMA2%B) by serum 25(OH)D quartiles (*n* = 256), 1 ng/mL 25-hydroxyvitamin D being equivalent to 0.4 nmol/L. Bars represent least-square means (with whisker for standard error) from multivariable linear regression models. Models were adjusted for age, sex, MVPA, smoking status (non, past, and current smoker) and body mass index. 25(OH)D, 25-hydroxyvitamin D; MVPA, moderate to vigorous physical activity (min/day).

For beta cell function (Table 3, Figure 2), adjusting for age, sex, smoking status, BMI, and physical activity, participants in the second serum 25(OH)D quartile had a higher HOMA2%B (77.9% vs. 59.6%; *p* = 0.02) compared with participants in the highest serum 25(OH)D quartile, with an inverse linear relationship between 25(OH)D quartiles and HOMA2%B (*p*_linear trend_ = 0.03).

**Figure 3:**
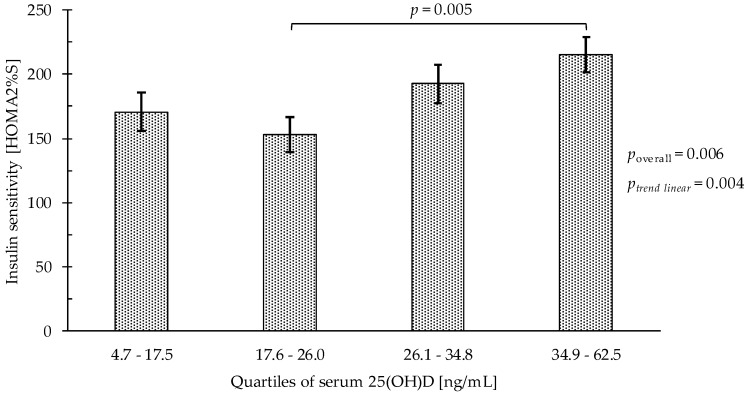
Insulin sensitivity (HOMA2%S) by serum 25(OH)D quartiles (*n* = 256), 1 ng/mL 25-hydroxyvitamin D being equivalent to 0.4 nmol/L. Bars represent least-square means (with whisker for standard error) from multivariable linear regression models. Models were adjusted for age, sex, MVPA, smoking status (non, past, and current smoker) and body mass index. 25(OH)D, 25-hydroxyvitamin D; MVPA, moderate to vigorous physical activity (min/day).

For insulin sensitivity (Table 3, Figure 3), adjusting for age, sex, smoking status, BMI, and physical activity, HOMA2%S of participants in the second serum 25(OH)D quartile was lower (152.8% vs. 215.3%; *p* = 0.005) compared with participants in the highest quartile. Moreover, HOMA2%S increased with higher quartiles of serum 25(OH)D (*p*_linear trend_ = 0.004).

For insulin resistance, adjusting for age, sex, smoking status, BMI, and physical activity, HOMA2-IR did not differ significantly between serum 25(OH)D quartiles.

### 3.5. Serum 25(OH)D Status and Prevalence of Insulin Resistance and Diabetes

For insulin resistance (Table 4), in unadjusted analysis, the prevalence differed significantly between serum 25(OH)D quartiles (*p* = 0.05). Notably, in the lowest serum 25(OH)D quartile, prevalence of insulin resistance was 3.8 times higher than in the highest 25(OH)D quartile (23.8% vs. 6.3%, *p* = 0.01). Adjusted for age, sex, physical activity, smoking status, and BMI, the prevalence of insulin resistance did not differ significantly between serum 25(OH)D quartiles overall (*p* = 0.29). However, notably in the second serum 25(OH)D quartile, there was a signal that the prevalence of insulin resistance may be higher than in the highest 25(OH)D quartile (*p* = 0.06).

For prevalence of diabetes (Table 4), in unadjusted analysis, the prevalence did not differ significantly between serum 25(OH)D quartiles (*p* = 0.57).

## 4. Discussion

In this cross-sectional, secondary analysis using baseline data of a completed RCT among 271 senior men and women, we found that a higher 25(OH)D status may be associated with lower body fat mass, higher insulin sensitivity, and better beta cell function, independent of BMI and other risk factors of diabetes including age, sex, smoking status, and an objective measure of physical activity. Alternatively, despite our adjustment for BMI, we cannot exclude that weight and fatness may influence both 25(OH)D level and glucose metabolism. Notably, for muscle mass, we did not find an association with 25(OH)D status, possibly because of an extended period of decreased mobility in our participants due to total knee replacement for severe knee osteoarthritis in our study.

Our result that lower serum 25(OH)D concentration was associated with higher body fat mass is in line with several other observational studies [8,30,31,32] among middle-aged and older adults. By quartiles of 25(OH)D serum concentrations, we documented that fat mass, was highest in the lowest 25(OH)D quartile (≤17.5 ng/mL) and lowest in the highest 25(OH)D quartile (≥34.9 ng/mL). Similarly, better insulin sensitivity and beta cell function were associated with the highest serum 25(OH)D quartile (≥34.9 ng/mL). A vitamin D status in the lowest or second quartile for 25(OH)D status (≤17.6–26.0 ng/mL) was associated with a least desirable glucose metabolism.

To our knowledge, this is the first study to explore the association of 25(OH)D status and glucose metabolism among relatively healthy European senior adults. Among our study participants, unselected for their body mass index, we found that in unadjusted analysis, prevalence of insulin resistance (HOMA2 IR ≥ 1.8) was 3.8-fold higher among participants in the lowest compared with the highest 25(OH)D quartile. However, after adjustment for age, sex, physical activity, smoking status, and BMI, this observation only approached statistical significance (*p* = 0.06). Previous studies performed mostly among adults between the ages 53 to 75 years, reported an inverse association between blood 25(OH)D concentration and HOMA IR in men only [33], in both men and women [34,35,36], or not at all [37,38]. Our findings on prevalence of insulin resistance being lowest in the highest quartile of 25(OH)D status among men and women (mean age 70.3 years), would fit well with the observed significant association of better insulin sensitivity, better beta cell function, and lower fat mass with higher 25(OH)D status in our study participants. The loss of statistical significance after adjusting for potential confounders could be due to the rather small number (*n* = 45) of insulin-resistant individuals among our participants, also preventing our analyses to extend to diabetes prevalence.

While our results demonstrate a relatively consistent association between 25(OH)D status and several components of body composition, including fat mass and glucose metabolism, the underlying premise that vitamin D status drives body composition cannot be established due to the cross-sectional study design. Alternatively, a higher body weight and greater body fatness may influence both 25(OH)D level and glucose metabolism. In fact, it has been well documented that overweight individuals have lower blood concentration of 25(OH)D than normal-weight individuals [39]. This cannot be explained only by lower vitamin D intake [40] or less sun exposure among heavier people [41], and probably reflects the larger tissue pool size over which the metabolite is distributed [42]. Also, it has been hypothesized that vitamin D and its metabolites are sequestered in fat tissue [43]. We tried to adjust for this concern by including BMI as an adjustment in our analyses, but still cannot exclude this alternative association.

Contrary to our expectations, we did not find an association between serum 25(OH)D concentration and muscle mass, neither among men nor among women. Prior studies largely support a positive association between higher serum 25(OH)D concentration and better muscle mass in senior adults [19,32,44,45,46], however not consistently so [47]. A possible explanation for missing such an association in our study may be the fact that our participants had undergone total knee replacement in the last 6 to 10 weeks prior to assessment. This may have masked an association between 25(OH)D status and muscle mass due to extended periods of pain and surgery-related reduced mobility imposed by their symptomatic knee osteoarthritis.

Our study has several strengths. First, to our knowledge, this is the first study to explore the association of 25(OH)D status and several components of body composition and glucose metabolism in a larger sample of relatively healthy European senior adults age 60 years and older. Second, our findings on fat mass and several key components of glucose metabolism (significant for insulin sensitivity and beta cell function, and a non-significant signal for insulin resistance) show a consistent association with 25(OH)D status D. Third, our findings were independent of sex and other key covariates that may influence body composition, including an objective high-quality measure of physical activity. Finally, for measurements of fat and muscle mass, we used DXA, which is considered the gold standard of these measures.

Our study also has limitations. The cross-sectional study design does not allow to draw causal relationships as addressed above. Further, our study may not be generalizable to adults younger than 60 years of age or frailer/less healthy older adults, since information on history of chronic diseases was not gathered. Also, we may have missed an association between muscle mass and 25(OH)D status due to the specific selection of participants mentioned above. Finally, the diet of the participants could have been a considerable confounder. Participants with more fat mass are likely to have a different diet than those with less fat mass, a factor that we were not able to adjust for.

In summary, our study documents a consistent association between 25(OH)D serum concentration and several components of body composition and glucose metabolism in relatively healthy senior adults. A 25(OH)D status in the highest 25(OH)D quartile range (≥ 34.9 ng/mL; range 34.9–62.5) was associated with lower fat mass, better insulin sensitivity, better beta cell function, and a non-significant signal of a lower prevalence of insulin resistance. On the other hand, for fat mass, a vitamin D-deficient state represented by the lowest quartile of 25(OH)D status (≤17.5 ng/mL; range 4.7–17.5), and for glucose metabolism, a vitamin D status represented in the lowest or second (17.6–26.0 ng/mL) quartile of 25(OH)D was associated with the highest fat mass and less advantageous glucose metabolism, respectively.

In conclusion, our cross-sectional study provides consistent signals that a better 25(OH)D status may be associated with a healthier glucose metabolism and body composition in relatively healthy adults age 60 years and older, and independent of BMI and other risk factors for diabetes. Further validation of our findings is needed in longitudinal studies.

## Figures and Tables

**Table 1:** Characteristics of participants of the Zurich Knee Osteoarthritis trial by sex.

Variables	Unit	Men	Women	Sex Difference (*p*)	Total Participants
Subjects	[*n* (%)]	126 (46.5)	145 (53.5)	0.25	271
Age	[year]	70.3 (6.9)	70.3 (6.0)	0.94	70.3 (6.4)
Height	[m]	1.8 (0.1)	1.6 (0.1)	<0.0001	1.7 (0.1)
Weight	[kg]	85.3 (12.4)	70.7 (11.3)	<0.0001	77.5 (13.9)
BMI	[kg/m^2^]	27.6 (3.8)	26.9 (4.1)	0.14	27.2 (3.9)
Not overweight (BMI <25 kg/m^2^)	[*n* (%)]	30 (23.8)	54 (37.2)	0.002	84 (31.0)
Overweight (BMI ≥25–29.99 kg/m^2^)		76 (60.3)	56 (38.6)		132 (48.7)
Obese (BMI ≥30)		20 (15.9)	35 (24.2)		55 (20.3)
Physical activity	[min MVPA/day]	45.1 (22.8)	38.0 (21.2)	0.01	41.3 (22.2)
Smoking status					
Non-smoker	[*n* (%)]	38 (30.2)	98 (67.6)	<0.0001	136 (50.2)
Past smoker		69 (54.7)	41 (28.3)		110 (40.6)
Current smoker		19 (15.1)	6 (4.1)		25 (9.2)
Baseline 25-hydroxyvitamin D	[ng/mL]	26.3 (11.3)	28.2 (13.3)	0.20	27.3 (12.4)
<10	[*n* (%)]	7 (5.6)	10 (6.9)	0.65	17 (6.3)
<20		38 (30.2)	47 (32.4)	0.69	85 (31.4)
Glucose metabolism					
Glucose	[mmol/L]	5.9 (1.4)	5.3 (0.6)	<0.0001	5.6 (1.1)
Insulin	[mU/L]	9.0 (8.8)	7.0 (6.1)	0.03	7.9 (7.5)
Beta cell function	[HOMA2%B]	75.3 (38.5)	71.7 (38.2)	0.45	73.3 (38.3)
Insulin sensitivity	[HOMA2%S]	143.3 (105.9)	195.7 (129.5)	0.0005	171.1 (121.6)
Insulin resistance	[HOMA2 IR]	1.2 (1.2)	0.9 (0.7)	0.005	1.1 (1.0)
Prevalent diabetes	[*n* (%)]	10 (8.0)	0 (0)	0.0004	10 (3.7)
DXA variables					
Total lean mass	[kg]	59.3 (6.8)	42.0 (4.8)	<0.0001	50.1 (10.4)
	[%]	70.0 (4.6)	60.0 (5.3)	<0.0001	64.7 (7.0)
Lean mass index	[kg/m^2^]	19.2 (1.8)	16.0 (1.6)	<0.0001	17.5 (2.3)
Appendicular lean mass	[kg]	26.3 (5.1)	17.7 (2.4)	<0.0001	21.7 (5.8)
	[%]	31.0 (4.5)	25.3 (2.6)	<0.0001	27.9 (4.6)
Appendicular lean mass index	[kg/m^2^]	8.5 (1.5)	6.7 (0.8)	<0.0001	7.6 (1.5)
Total fat mass	[kg]	23.2 (7.0)	27.1 (7.7)	<0.0001	25.3 (7.6)
	[%]	26.7 (4.9)	37.7 (5.5)	<0.0001	32.6 (7.6)
Fat mass index	[kg/m^2^]	7.5 (2.3)	10.3 (2.9)	<0.0001	9.0 (3.0)

Data (*n* = 271) are crude means (± SD) or *n* (%). Differences between men and women were assessed by using Student’s *t* test for continuous variables and a χ^2^ test for categorical variables. *p* values are two-sided; statistical significance is set at *p* < 0.05. 1 ng/mL 25-hydroxyvitamin D being equivalent to 0.4 nmol/L. Abbreviations: 25(OH)D, 25-hydroxyvitamin D; BMI, Body Mass Index; DXA, dual-energy x-ray absorptiometry; MVPA, moderate to vigorous physical activity; HOMA, homeostatic model assessment.

**Table 2:** Body composition parameters by 25-hydroxyvitamin D [ng/mL] quartiles (Q_1_–Q_4_) for total participants.

Parameter	Q_1_ (4.7–17.5) (*n* = 67)	Q_2_ (17.6–26.0) (*n* = 69)	Q_3_ (26.1–34.8) (*n* = 67)	Q_4_ (34.9–62.5) (*n* = 68)
Fat mass index [kg/m^2^]				
LSM (95% CI)	9.31 (8.77, 9.84)	8.69 (8.17, 9.20)	8.40 (7.87, 8.94)	8.37 (7.88, 8.87)
*p* ^†^	Ref	0.28	0.049	0.04
*p_overall_* ^‡^	0.03			
*p_trend linear_*	0.65			
Appendicular lean mass index [kg/m^2^]				
LSM (95% CI)	7.74 (7.54, 7.95)	7.68 (7.48, 7.87)	7.80 (7.59, 8.00)	7.54 (7.35, 7.73)
*p* ^†^	Ref	0.96	0.98	0.46
*p_overall_* ^‡^	0.26			
*p_trend linear_*	0.06			

Data (*n* = 271 for FMI; *n* = 270 for ALMI) are LSM (95% CI) from multivariable linear regression models. Models were adjusted for age, sex, MVPA, smoking status (non, past, and current smoker) and BMI (model for FMI was adjusted for lean mass index instead of BMI). *p* for a linear trend across the 25(OH)D quartiles was calculated from linear regression models using the median values of individual 25(OH)D quartiles as a continuous variable. *p* values are two-sided. Statistical significance is set at *p* < 0.05. 1 ng/mL 25-hydroxyvitamin D being equivalent to 0.4 nmol/L. Abbreviations: Q_1_–Q_4_, quartiles of serum 25-hydroxyvitamin D concentration [ng/mL], 25(OH)D, 25-hydroxyvitamin D; MVPA, moderate to vigorous physical activity (min/day); FMI, fat mass index; LSM, least-square means; Ref, reference quartile with each *p*-value referring to this quartile. ^†^ *p* value for the difference between two 25(OH)D quartiles with the first quartile being the reference. ^‡^ *p* value for the overall differences among all 25(OH)D quartiles.

**Table 3:** Parameters of glucose metabolism by 25-hydroxyvitamin D [ng/mL] quartiles (Q_1_–Q_4_) for total participants.

Parameter	Q_1_ (4.7–17.5)	Q_2_ (17.6–26.0)	Q_3_ (26.1–34.8)	Q_4_ (34.9–62.5)
Fasting glucose [mmol/L]	(*n* = 63)	(*n* = 66)	(*n* = 64)	(*n* = 67)
LSM (95% CI)	5.46 (5.17, 5.75) ^a^	5.47 (5.21,5.74) ^a^	5.76 (5.48, 6.05) ^a^	5.54 (5.28, 5.81) ^a^
*p* ^†^	Ref	1.00	0.36	0.97
*p_overall_* ^‡^	0.31			
*p_trend linear_*	0.44			
Fasting insulin [mU/L]	(*n* = 66)	(*n* = 69)	(*n* = 66)	(*n* = 56)
LSM (95% CI)	7.40 (5.57, 9.23) ^a^	8.21 (6.49, 9.93) ^a^	8.33 (6.51, 10.16) ^a^	5.64 (3.93, 7.35) ^a^
*p* ^†^	Ref	0.90	0.86	0.48
*p_overall_* ^‡^	0.09			
*p_trend linear_*	0.13			
Beta cell function [HOMA2%B]	(*n* = 63)	(*n* = 66)	(*n* = 63)	(*n* = 64)
LSM (95% CI)	70.6 (60.7, 80.5) ^ab^	77.9 (68.7, 87.1) ^a^	69.8 (60.0, 79.5) ^ab^	59.6 (50.5, 68.7) ^b^
*p* ^†^	Ref	0.65	1.00	0.34
*p_overall_* ^‡^	0.04			
*p_trend linear_*	0.03			
Insulin sensitivity [HOMA2%S]	(*n* = 63)	(*n* = 66)	(*n* = 63)	(*n* = 64)
LSM (95% CI)	170.7 (141.1, 200.2) ^ab^	152.8 (125.4, 180.2) ^a^	192.6 (163.5, 221.8) ^ab^	215.3 (188.2, 242.5) ^b^
*p* ^†^	Ref	0.78	0.66	0.10
*p_overall_* ^‡^	0.01			
*p_trend linear_*	0.004			

Data (*n* = 260 for fasting glucose; *n* = 266 for fasting insulin; *n* = 256 for all HOMA2 values) are LSM (95% CI) from multivariable linear regression models. Models were adjusted for age, sex, MVPA, smoking status (non, past, and current smoker) and body mass index. *p* for a linear trend across the serum 25(OH)D quartiles was calculated from linear regression models using the median values of individual 25(OH)D quartiles as a continuous variable. LSM with different superscript letters (a b) are significantly different from each other. *p* values are two-sided. Statistical significance is set at *p* < 0.05. 1 ng/mL 25-hydroxyvitamin D being equivalent to 0.4 nmol/L. Abbreviations: Q_1_–Q_4_, quartiles of serum 25-hydroxyvitamin D concentration [ng/mL], 25(OH)D, 25-hydroxyvitamin D; LSM, least-square means; MVPA, moderate to vigorous physical activity (min/d); HOMA, homeostatic model assessment. Ref, reference quartile with each *p*-value referring to this quartile. ^†^ *p* value for the difference between two 25(OH)D quartiles with the first quartile being the reference. ^‡^ *p* value for the overall differences among all 25(OH)D quartiles.

**Table 4:** Prevalence of insulin resistance and diabetes by 25-hydroxyvitamin D [ng/mL] quartiles (Q_1_–Q_4_) *.

	Q_1_ (4.7–17.5)	Q_2_ (17.6–26.0)	Q_3_ (26.1–34.8)	Q_4_ (34.9–62.5)	Total Participants
Unadjusted model					
Insulin resistance (HOMA2 IR ≥ 1.8)					
*n* (%)	15 (23.8)	14 (21.2)	12 (19.0)	4 (6.3)	45
*p_overall_* ^†^	0.05				
*p* ^‡^	0.01				
*p* ^§^	0.02				
Diabetes (fasting glucose >7.0 mmol/L)					
*n* (%)	3 (4.8)	2 (3.0)	4 (6.3)	1 (1.5)	10
*p_overall_* ^†^	0.57				
*p* ^‡^	0.31				
*p* ^§^	0.56				
Adjusted model					
Insulin resistance (HOMA2 IR ≥ 1.8)					
% (95% CI)	9.4 (3.8, 21.2)	14.6 (7.2, 27.1)	11.9 (5.3, 24.4)	4.8 (1.6, 13.7)	
*p_overall_* ^†^	0.29				
*p* ^‡^	0.29				
*p* ^§^	0.06				

Data (*n* = 256 for HOMA2 IR or *n* = 260 for diabetes) are *n* (%) and % (95% CI) for the prevalence of insulin resistance (HOMA2 IR ≥ 1.8) and diabetes (fasting blood glucose >7.0 mmol/L) by quartiles of serum 25-hydroxyvitamin D derived from generalized linear models (logit link function) unadjusted and adjusted for age, sex, MVPA, smoking status (non, past, and current smoker) and body mass index. *p* values are two-sided and *p* < 0.05 was considered statistically significant. 1 ng/mL 25-hydroxyvitamin D being equivalent to 0.4 nmol/L. Abbreviations: Q_1_–Q_4_, quartiles of serum 25-hydroxyvitamin D concentration [ng/mL], MVPA, moderate to vigorous physical activity (min/day); HOMA, homeostatic model assessment.* For insulin resistance: Q_1_ (*n* = 63), Q_2_ (*n* = 66), Q_3_ (*n* = 63), Q_4_ (*n* = 64); for diabetes: Q_1_ (*n* = 63), Q_2_ (*n* = 66), Q_3_ (*n* = 64), Q_4_ (*n* = 67) ^†^ *p* value for the overall difference between quartiles. ^‡^ *p* value for the difference between Q_1_ and Q_4_^§^ *p* value for the difference between Q_2_ and Q_4_.

## References

[1] Roubenoff R. (2004). Sarcopenic obesity: The confluence of two epidemics. Obes. Res..

[2] Baumgartner R.N. (2000). Body composition in healthy aging. Ann. N. Y. Acad. Sci..

[3] Cleasby M.E., Jamieson P.M., Atherton P.J. (2016). Insulin resistance and sarcopenia: Mechanistic links between common co-morbidities. J. Endocrinol..

[4] Baumgartner R.N., Wayne S.J., Waters D.L., Janssen I., Gallagher D., Morley J.E. (2004). Sarcopenic obesity predicts instrumental activities of daily living disability in the elderly. Obes. Res..

[5] Prado C.M., Wells J.C., Smith S.R., Stephan B.C., Siervo M. (2012). Sarcopenic obesity: A Critical appraisal of the current evidence. Clin. Nutr..

[6] Cashman K.D., Dowling K.G., Skrabakova Z., Gonzalez-Gross M., Valtuena J., De Henauw S., Moreno L., Damsgaard C.T., Michaelsen K.F., Molgaard C. (2016). Vitamin D deficiency in Europe: Pandemic?. Am. J. Clin. Nutr..

[7] Bischoff-Ferrari H. (2013). Relevance of vitamin D in bone and muscle health of cancer patients. Anticancer Agents Med. Chem..

[8] Oliai Araghi S., van Dijk S.C., Ham A.C., Brouwer-Brolsma E.M., Enneman A.W., Sohl E., Swart K.M., van der Zwaluw N.L., van Wijngaarden J.P., Dhonukshe-Rutten R.A. (2015). BMI and body fat mass is inversely associated with Vitamin D levels in older individuals. J. Nutr. Health Aging.

[9] Shantavasinkul P.C., Phanachet P., Puchaiwattananon O., Chailurkit L.O., Lepananon T., Chanprasertyotin S., Ongphiphadhanakul B., Warodomwichit D. (2015). Vitamin D status is a determinant of skeletal muscle mass in obesity according to body fat percentage. Nutrition.

[10] Tosunbayraktar G., Bas M., Kut A., Buyukkaragoz A.H. (2015). Low serum 25(OH)D levels are associated to higher BMI and metabolic syndrome parameters in adult subjects in Turkey. Afr. Health Sci..

[11] Bischoff H.A., Borchers M., Gudat F., Duermueller U., Theiler R., Stahelin H.B., Dick W. (2001). In situ detection of 1,25-dihydroxyvitamin D3 receptor in human skeletal muscle tissue. Histochem. J..

[12] Bischoff-Ferrari H.A., Borchers M., Gudat F., Durmuller U., Stahelin H.B., Dick W. (2004). Vitamin D receptor expression in human muscle tissue decreases with age. J. Bone Miner Res..

[13] Ceglia L., da Silva Morais M., Park L.K., Morris E., Harris S.S., Bischoff-Ferrari H.A., Fielding R.A., Dawson-Hughes B. (2010). Multi-step immunofluorescent analysis of vitamin D receptor loci and myosin heavy chain isoforms in human skeletal muscle. J. Mol. Histol..

[14] Ceglia L., Niramitmahapanya S., da Silva Morais M., Rivas D.A., Harris S.S., Bischoff-Ferrari H., Fielding R.A., Dawson-Hughes B. (2013). A randomized study on the effect of vitamin D(3) supplementation on skeletal muscle morphology and vitamin D receptor concentration in older women. J. Clin. Endocrinol. Metab..

[15] Ding C., Gao D., Wilding J., Trayhurn P., Bing C. (2012). Vitamin D signalling in adipose tissue. Br. J. Nutr..

[16] Shi H., Norman A.W., Okamura W.H., Sen A., Zemel M.B. (2001). 1alpha,25-Dihydroxyvitamin D3 modulates human adipocyte metabolism via nongenomic action. FASEB J..

[17] Mitri J., Dawson-Hughes B., Hu F.B., Pittas A.G. (2011). Effects of vitamin D and calcium supplementation on pancreatic beta cell function, insulin sensitivity, and glycemia in adults at high risk of diabetes: The Calcium and Vitamin D for Diabetes Mellitus (CaDDM) randomized controlled trial. Am. J. Clin. Nutr..

[18] Pittas A.G., Harris S.S., Stark P.C., Dawson-Hughes B. (2007). The effects of calcium and vitamin D supplementation on blood glucose and markers of inflammation in nondiabetic adults. Diabetes Care.

[19] Ko M.J., Yun S., Oh K., Kim K. (2015). Relation of serum 25-hydroxyvitamin D status with skeletal muscle mass by sex and age group among Korean adults. Br. J. Nutr..

[20] Visser M., Deeg D.J., Lips P. (2003). Longitudinal Aging Study A: Low vitamin D and high parathyroid hormone levels as determinants of loss of muscle strength and muscle mass (sarcopenia): The Longitudinal Aging Study Amsterdam. J. Clin. Endocrinol. Metab..

[21] Al-Shoha A., Qiu S., Palnitkar S., Rao D.S. (2009). Osteomalacia with bone marrow fibrosis due to severe vitamin D deficiency after a gastrointestinal bypass operation for severe obesity. Endocr. Pract..

[22] Bischoff-Ferrari H.A., Dietrich T., Orav E.J., Hu F.B., Zhang Y., Karlson E.W., Dawson-Hughes B. (2004). Higher 25-hydroxyvitamin D concentrations are associated with better lower-extremity function in both active and inactive persons aged > or = 60 year. Am. J. Clin. Nutr..

[23] Soares M.J., Chan She Ping-Delfos W., Ghanbari M.H. (2011). Calcium and vitamin D for obesity: A review of randomized controlled trials. Eur. J. Clin. Nutr..

[24] Maunsell Z., Wright D.J., Rainbow S.J. (2005). Routine isotope-dilution liquid chromatography-tandem mass spectrometry assay for simultaneous measurement of the 25-hydroxy metabolites of vitamins D2 and D3. Clin. Chem..

[25] Saenger A.K., Laha T.J., Bremner D.E., Sadrzadeh S.M. (2006). Quantification of serum 25-hydroxyvitamin D(2) and D(3) using HPLC-tandem mass spectrometry and examination of reference intervals for diagnosis of vitamin D deficiency. Am. J. Clin. Pathol..

[26] The National Institute of Standards and Technology (NIST), The National Institutes of Health (NIH) Vitamin D Metabolites Quality Assurance Program. http://www.nist.gov/mml/analytical/vitdqap.cfm.

[27] Resnick B., Nahm E.S., Orwig D., Zimmerman S.S., Magaziner J. (2001). Measurement of activity in older adults: Reliability and validity of the Step Activity Monitor. J. Nurs. Meas..

[28] White D.K., Tudor-Locke C., Zhang Y., Fielding R., LaValley M., Felson D.T., Gross K.D., Nevitt M.C., Lewis C.E., Torner J. (2014). Daily walking and the risk of incident functional limitation in knee osteoarthritis: An observational study. Arthritis Care Res..

[29] Geloneze B., Vasques A.C., Stabe C.F., Pareja J.C., Rosado L.E., Queiroz E.C., Tambascia M.A., Investigators B. (2009). HOMA1-IR and HOMA2-IR indexes in identifying insulin resistance and metabolic syndrome: Brazilian Metabolic Syndrome Study (BRAMS). Arq. Bras. Endocrinol. Metabol..

[30] Jungert A., Neuhauser-Berthold M. (2015). Sex-specific determinants of serum 25-hydroxyvitamin D3 concentrations in an elderly German cohort: A cross-sectional study. Nutr. Metab. (Lond).

[31] Moschonis G., Tanagra S., Koutsikas K., Nikolaidou A., Androutsos O., Manios Y. (2009). Association between serum 25-hydroxyvitamin D levels and body composition in postmenopausal women: The postmenopausal Health Study. Menopause.

[32] Seo J.A., Cho H., Eun C.R., Yoo H.J., Kim S.G., Choi K.M., Baik S.H., Choi D.S., Park M.H., Han C. (2012). Association between visceral obesity and sarcopenia and vitamin D deficiency in older Koreans: The Ansan Geriatric Study. J. Am. Geriatr. Soc..

[33] Sun X., Cao Z.B., Tanisawa K., Ito T., Oshima S., Higuchi M. (2014). The relationship between serum 25-hydroxyvitamin D concentration, cardiorespiratory fitness, and insulin resistance in Japanese men. Nutrients.

[34] Esteghamati A., Aryan Z., Esteghamati A., Nakhjavani M. (2015). Vitamin D deficiency is associated with insulin resistance in nondiabetics and reduced insulin production in type 2 diabetics. Horm. Metab. Res..

[35] Jiang H., Peng S. (2014). The relationship between serum vitamin D and HOMA-IR in overweight elderly patients. Int. J. Cardiol..

[36] Liu E., Meigs J.B., Pittas A.G., McKeown N.M., Economos C.D., Booth S.L., Jacques P.F. (2009). Plasma 25-hydroxyvitamin D is associated with markers of the insulin resistant phenotype in nondiabetic adults. J. Nutr..

[37] Brouwer-Brolsma E.M., Feskens E.J., Steegenga W.T., de Groot L.C. (2013). Associations of 25-hydroxyvitamin D with fasting glucose, fasting insulin, dementia and depression in European elderly: The SENECA study. Eur. J. Nutr..

[38] Song B.M., Kim H.C., Choi D.P., Oh S.M., Suh I. (2014). Association between serum 25-hydroxyvitamin D level and insulin resistance in a rural population. Yonsei Med. J..

[39] Yetley E.A. (2008). Assessing the vitamin D status of the US population. Am. J. Clin. Nutr..

[40] Hypponen E., Power C. (2007). Hypovitaminosis D in British adults at age 45 year: Nationwide cohort study of dietary and lifestyle predictors. Am. J. Clin. Nutr..

[41] Harris S.S., Dawson-Hughes B. (2007). Reduced sun exposure does not explain the inverse association of 25-hydroxyvitamin D with percent body fat in older adults. J. Clin. Endocrinol. Metab..

[42] Drincic A.T., Armas L.A., Van Diest E.E., Heaney R.P. (2012). Volumetric dilution, rather than sequestration best explains the low vitamin D status of obesity. Obesity.

[43] Wortsman J., Matsuoka L.Y., Chen T.C., Lu Z., Holick M.F. (2000). Decreased bioavailability of vitamin D in obesity. Am. J. Clin. Nutr..

[44] Marantes I., Achenbach S.J., Atkinson E.J., Khosla S., Melton L.J., Amin S. (2011). Is vitamin D a determinant of muscle mass and strength?. J. Bone Miner Res..

[45] Park S., Ham J.O., Lee B.K. (2014). A positive association of vitamin D deficiency and sarcopenia in 50 year old women, but not men. Clin. Nutr..

[46] Tieland M., Brouwer-Brolsma E.M., Nienaber-Rousseau C., van Loon L.J., De Groot L.C. (2013). Low vitamin D status is associated with reduced muscle mass and impaired physical performance in frail elderly people. Eur. J. Clin. Nutr..

[47] Gumieiro D.N., Murino Rafacho B.P., Buzati Pereira B.L., Cavallari K.A., Tanni S.E., Azevedo P.S., Polegato B.F., Mamede Zornoff L.A., Dinhane D.I., Innocenti Dinhane K.G. (2015). Vitamin D serum levels are associated with handgrip strength but not with muscle mass or length of hospital stay after hip fracture. Nutrition.

